# Towards Understanding the Relationship Between ER Stress and Unfolded Protein Response in Amyotrophic Lateral Sclerosis

**DOI:** 10.3389/fnagi.2022.892518

**Published:** 2022-06-15

**Authors:** Chenxuan Zhao, Yong Liao, Abdul Rahaman, Vijay Kumar

**Affiliations:** ^1^School of Engineering, College of Technology and Business, Guangzhou, China; ^2^Center of Scientific Research, Maoming People’s Hospital, Maoming, China; ^3^School of Food Science and Engineering, South China University of Technology, Guangzhou, China; ^4^Amity Institute of Neuropsychology & Neurosciences (AINN), Amity University, Noida, India

**Keywords:** ER stress, UPR, ALS, TDP-43, pharmacological modulator

## Abstract

Biological stress due to the aberrant buildup of misfolded/unfolded proteins in the endoplasmic reticulum (ER) is considered a key reason behind many human neurodegenerative diseases. Cells adapted to ER stress through the activation of an integrated signal transduction pathway known as the unfolded protein response (UPR). Amyotrophic lateral sclerosis (ALS) is a neurodegenerative disease characterized by degeneration of the motor system. It has largely been known that ER stress plays an important role in the pathogenesis of ALS through the dysregulation of proteostasis. Moreover, accumulating evidence indicates that ER stress and UPR are important players in TDP-43 pathology. In this mini-review, the complex interplay between ER stress and the UPR in ALS and TDP-43 pathology will be explored by taking into account the studies from *in vitro* and *in vivo* models of ALS. We also discuss therapeutic strategies to control levels of ER stress and UPR signaling components that have contrasting effects on ALS pathogenesis.

## Introduction

Amyotrophic lateral sclerosis (ALS), the most common type of motor neuron disease, is characterized by the progressive degeneration of both lower and upper motor neurons and eventually leads to death due to respiratory failure, typically within 2–5 years of symptom onset (van Es et al., 2017). While the majority of cases are sporadic (sALS), approximately 10% of ALS is inherited, usually in an autosomal dominant fashion (fALS). The proposed pathomechanisms of ALS include oxidative stress, dysfunction in mitochondria and axonal transport, excitotoxicity, damage to protein homeostasis, etc. (Ferraiuolo et al., 2011; Kumar et al., 2016a; Tsai and Manley, 2021). Studies of fALS cases have revealed several ALS-associated genes (Cooper-Knock et al., 2021; Shatunov and Al-Chalabi, 2021) which are involved in several important cellular and biological processes. Many of the fALS- genes have also been involved in different facets of the proteostasis network. Abnormal accumulation of misfolded or aggregated proteins to proteinaceous inclusions represents a common unifying pathological feature of ALS (Lin et al., 2017; Webster et al., 2017; Cicardi et al., 2021). These intracellular inclusions are found in both degenerating neurons and surrounding glia (Nishihira et al., 2009; Dugger and Dickson, 2017) as well as present in the different regions of the brain including the spinal cord, cerebellum, brainstem, hippocampus, and the frontal and temporal lobes (Al-Chalabi et al., 2012). The most predominant inclusions observed in the motor neurons are of ubiquitinated proteins (Neumann et al., 2006), thus indicating the defects in protein homeostasis (Blokhuis et al., 2013). Ubiquitinated protein inclusions of Tar DNA-binding protein of 43 kDa (TDP-43) are positive for the majority of ALS cases (Arai et al., 2006; Neumann et al., 2006). TDP-43 was also identified as the pathological protein in a subset of neurodegenerative diseases commonly known as TDP-43 proteinopathies (Tziortzouda et al., 2021). In these diseases, the loss of nuclear TDP-43 and cytoplasmic inclusions of TDP-43 result in either loss or gain-of-function within neurons and affect several biological processes like autophagy, RNA synthesis, the ubiquitin-proteasome system, and axonal transport (Prasad et al., 2019). Several studies also indicate that endoplasmic reticulum (ER) stress plays a critical role in TDP-43 proteinopathies (Walker and Atkin, 2011; Walter and Ron, 2011; Walker et al., 2013; de Mena et al., 2021). Besides the presence of TDP-43 inclusions, inclusions for mutant Cu/Zn superoxide dismutase (SOD1) and fused in sarcoma protein (FUS) are also found in ALS patients (Mackenzie et al., 2007; Kwiatkowski et al., 2009; Vance et al., 2009). The other fALS-associated mutant proteins that aggregates are valosin-containing protein (VCP), dynactin-1 (DCTN1), optineurin (OPTN), and ubiquilin-2 (UBQLN2; Levy et al., 2006; Maruyama et al., 2010; Deng et al., 2011; Koppers et al., 2012). The characteristic observation of the protein aggregates is indicative of the breakdown of proteostasis in ALS. Consequently, a key proteostatic pathway identified as the Unfolded Protein Response (UPR) responds to the endoplasmic reticulum (ER) stress-induced protein aggregation by initiating either proadaptive and/or proapoptotic pathways. In this mini-review, we aim to provide insights into the complex interplay between ER stress, UPR, and TDP-43 in the context of ALS.

## ER Stress and UPR Pathway

**Figure 1:**
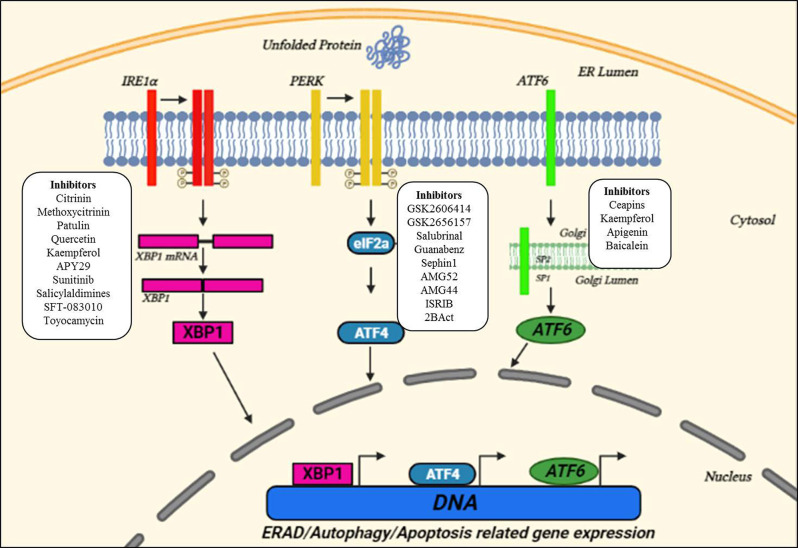
Unfolded protein response (UPR) pathways in the ER and small molecule interventions. Upon ER stress, the ER launches three adaptive pathways through IRE1α, PERK, and activating transcription factor 6 (ATF6), collectively called as the UPR, to restore proteostasis. All these three ER stress sensors activate signaling cascades, increasing protein-folding ability and decreasing ER stress. Small molecules targeting three key UPR signaling components are also indicated in the figure suggesting the sites for therapeutic interventions. Adapted and modified from Webster et al. (2017).

The ER is a membranous organelle involved in protein folding, post-translational modifications, and trafficking, and synthesizes about one-third of the total proteome (Schroder, 2008; Bernard-Marissal et al., 2015). Despite the quality control accomplished by ER chaperones to ensure proper folding and maturation of newly synthesized proteins, protein maturation mechanisms are sometimes perturbed which leads to the correct folding success in the cell to be under 20% (Kaushik and Cuervo, 2015). These misfolded/unfolded proteins are retained in the ER and are susceptible to undergoing proteasomal degradation through the ER-associated degradation (ERAD) pathway that recognizes, ubiquitinates, and relocates misfolded proteins to the cytosol for their degradation (Oakes and Papa, 2015). However, disruption of ER-calcium homeostasis, abnormal proteostasis, hypoxia, etc., decrease the protein-folding ability leading to ER stress (Lin et al., 2008). Upon detection of ER stress, the ER initiates UPR that decreases the burden of misfolded proteins and re-establishes proteostasis. This cellular response is achieved through coordinated transcriptional and translational activities, the increased expression of chaperones in the ERAD pathway, and a brief decrease in the protein flux entering the ER. The UPR has three proximal transmembrane protein sensors: Inositol-requiring kinase/endoribonuclease (IRE1), double-stranded RNA-activated protein kinase (PKR)-like ER kinase (PERK), and activating transcription factor 6 (ATF6) (Schroder, 2008; Hetz, 2012). Both IRE-1 and PERK are type I transmembrane proteins having kinase activity (Liu et al., 2002). On the other hand, ATF6 is a type II transmembrane protein, and its cytosolic domain can enter the nucleus and thus can activate UPR related genes (Haze et al., 1999). The UPR attempts to restore proteostasis by decreasing translation, increasing chaperones assisted protein folding, and up-regulation of ERAD and autophagy to get rid of misfolded proteins. If ER stress is brief, the UPR pathways work in parallel to organize a series of pro-adaptive cascades to restore proteostasis and the cell survives. However, in case of severe prolonged ER stress, the UPR activates the pro-apoptotic pathway (Figure 1; Hetz and Saxena, 2017; Hetz and Papa, 2018).

An immediate adaptive reaction to ER stress is started by translocation of ATF6 to the nucleus, where it modulates the genes involved in protein homeostasis (Haze et al., 1999). IRE1 by mediating the splicing of a transcription factor, X-Box-Binding protein 1 (XBP1), activates several genes of the protein homeostasis pathway along with the release of ERAD proteins (Acosta-Alvear et al., 2007). Whereas, PERK phosphorylates the eukaryotic initiation factor 2α (eIF2α), and thus decreases protein translation by overpowering a load of misfolded proteins (Harding et al., 1999). Also, eIF2α activates ATF4 which subsequently activates many UPR-targeted genes (Tabas and Ron, 2011). Ultimately, the UPR reduced the translation and increased the expression of genes of protein homeostasis including chaperones and ERAD proteins. Also, disruption of ER homeostasis leads to the activation of apoptotic pathways (Malhotra and Kaufman, 2007; Krebs et al., 2015).

## ER Stress and UPR in ALS

Long-term ER stress is a critical factor affecting cell survival in neurodegenerative diseases characterized by severe proteostatic imbalances (Scheper and Hoozemans, 2015). Many studies have reported the presence of ER stress in ALS and FTD patient’s tissue samples, as well as cellular and animal models of fALS genes like SOD1, VAPB, or FUS (Kikuchi et al., 2006; Gitcho et al., 2009; Chen et al., 2010; Farg et al., 2012). The involvement of the UPR pathway in ALS has been shown in ALS patients’ post-mortem spinal cord as well as in ALS mice (Ilieva et al., 2007; Atkin et al., 2008; Sasaki, 2010). Morphological alterations such as dilation and fragmentation of rough ER have been observed in sALS patients and the mutant SOD1^G93A^ mice (Oyanagi et al., 2008; Lautenschlaeger et al., 2012). Sasaki (2010) has observed the accumulation of misfolded proteins as a granular or amorphous material in the ER of sALS patients.

Misfolding and aberrant deposition of SOD1 are thought to be responsible for ER stress in ALS cases since mutant SOD1 is prone to misfold, co-localize with ER resident markers like glucose-related protein 78 (Grp78) and calnexin (Wate et al., 2005; Kikuchi et al., 2006). Further evidence for a close association between UPR and misfolded SOD1 deposition was provided by the findings that showed the upregulation and subsequent co-localization of an ER chaperone, protein disulphideisomerase (PDI) in ALS patients, and SOD1^G93A^ mice (Atkin et al., 2006). Also, increased amounts of phosphorylated PERK (Atkin et al., 2006, 2008; Saxena et al., 2009), and phosphorylated eIF2α (Saxena et al., 2009) have been reported inSOD1^G93A^ mice as well as in Neuro2a cells transfected with mutant SOD1.

Interestingly, ER stress and subsequent activation of the UPR components have been observed in the primary motor neurons exposed to the CSF of sALS patients (Vijayalakshmi et al., 2009, 2011). The CSF-induced ER stress and neurodegeneration appear to involve caspase-12 activation by the UPR pathway (Nakagawa et al., 2000; Martinez et al., 2010). Increased levels of ATF6 have been reported in ALS patients and SOD1^G93A^ mice (Atkin et al., 2006, 2008). Oh et al. (2008) have shown the cleavage and movement of ATF6 in Neuro2a cells transfected with mutant SOD1^G85R^. While, Hetz et al. (2008) showed that ATF6 knockdown in NSC-34 cells transfected with mutant SOD1 increases SOD1 aggregation. Also, ALS patients and ALS mice showed increased IRE1 expression before the onset of disease (Atkin et al., 2006, 2008).

Several studies showed the involvement of C9orf72 pathogenesis in the ER stress in cell culture, primary cortical neurons, and iPSC-derived human neurons (Zhang et al., 2014; Zhang Y. J. et al., 2018; Kramer et al., 2018; Wang et al., 2019). Also, ER stress inducer tunicamycin showed a dose-dependent increase in cell death in C9-ALS iPSC-derived motoneurons, indicating altered ER proteostasis (Haeusler et al., 2014). C9orf72-ALS transcriptome studies from the human cerebellum and frontal cortex showed the upregulation of UPR genes, indicating the activation of ER stress (Prudencio et al., 2015). Dafinca et al. (2016) showed improved ER stress along with altered mitochondrial morphology and membrane potential in an iPSC-derived motor neurons from C9-ALS patients. Furthermore, an RNA sequencing study of mouse neurons having poly (PR) dipeptide repeats showed the upregulation of genes involved in ER stress, indicating that poly (PR) activates the UPR pathway (Kramer et al., 2018).

In another study by Wang et al. (2019), synthetic poly (PR) induced ER stress and inhibited the UPR mediated cell survival. Moreover, ER stress also increased the RAN translation of the G4C2 expansion, and overexpression of the G4C2 repeats decreased the global translation while increasing the stress granules formation in an eIF2α-dependent manner (Green et al., 2017). Westergard et al. (2019) have also reported the increased RAN translation and ER stress due to excitotoxic stress and optogenetic neuronal stimulation of cortical and spinal motor neurons from a C9orf72 model with (G4C2)_188_ repeat expansion.

A recent post-mortem study in C9-FTD patients showed increased levels of phosphorylated PERK and eIF2α in the hippocampus of C9-FTD patients and the increased levels were significantly correlated with the presence of poly (PR) pathology (Gami-Patel et al., 2021).

Therefore, integrated stress response (ISR) activation initiated by cellular stresses triggers RAN translation in cells and neurons and indicates a feed-forward loop between poly (PR) formation and the associated stress (Green et al., 2017; Cheng et al., 2018; Sonobe et al., 2018; Westergard et al., 2019). In this context, Sidrauski et al. (2013) reported that a small molecule inhibitor of ISR, ISRIB reduced poly (PR) induced toxicity. Moreover, Glineburg et al. (2021) have shown that activation of ISR by sodium arsenite-induced G4C2 repeat foci in C9orf72 patient fibroblasts. Administration of ISRIB or GSK2606414, PERK inhibitor showed strong neuroprotection in a fly model of C9orf72 pathogenesis (Zhang K. et al., 2018). Similar results were also shown in iPSC-derived motoneurons from C9-ALS cases (Zhang K. et al., 2018).

Importantly, inhibition of PKR largely reduced poly (PR) accumulation and improved behavior in C9-ALS/FTD transgenic mice (Zu et al., 2020). Overall, accumulating evidences indicate that inhibition of eIF2α phosphorylation or its downstream effects has neuroprotective consequences in the context of C9-ALS/FTD pathogenesis.

Similarly, the ER stress increased in NSC-34 cells with mutant FUS and in primary neurons expressing C9orf72-linked poly (GA) dipeptide repeats (Farg et al., 2012; Zhang et al., 2014).

**Figure 2:**
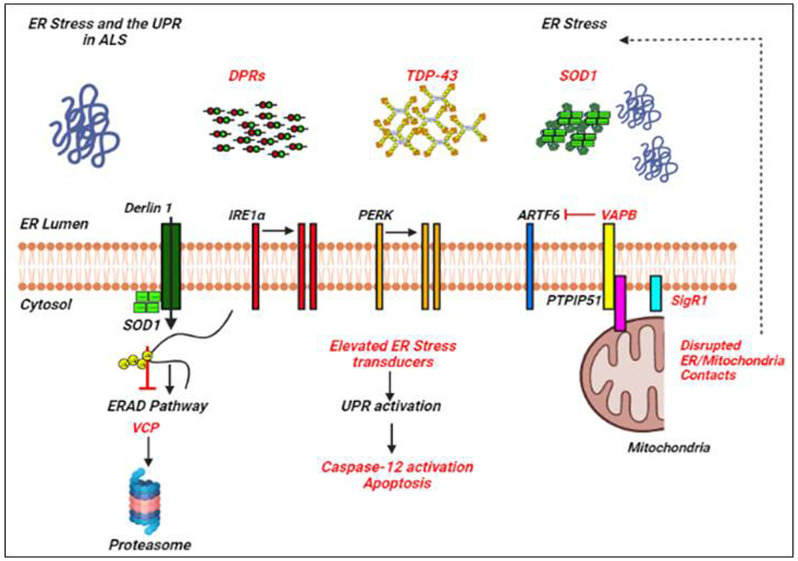
ER stress and the UPR in amyotrophic lateral sclerosis (ALS). Examples of ALS-linked genes involved in ER stress and the UPR are shown in red text. ALS-associated defects to the UPR are also mentioned in red text. Adapted from Webster et al. (2017).

Many of the ALS proteins significantly affect the UPR and/or proteostasis pathways as shown by different studies (Lin et al., 2017; Webster et al., 2017; Dafinca et al., 2021; Figure 2). ALS mutant SOD1 interacts with Derlin-1, an ER protein crucial for the ERAD pathway, and thus alters the ERAD which induces ER stress (Nishitoh et al., 2008). Vesicle-associated membrane protein-associated protein B (VAPB) is an important ER protein which is involved in the UPR activation through the IRE1 and ATF6 pathways, and mutations in VAPB cause ALS8 (Gkogkas et al., 2008; Suzuki et al., 2009). Overexpressed WT and mutant VAPB^P56S^ interact strongly with ATF6 and decrease the transcription of XBP1, thus inducing ER stress (Suzuki et al., 2009). Indeed, knockdown of VAPB has been shown to inhibit the IRE1/XBP1 pathway, VAPB is thus involved in the activation of UPR in response to ER stress (Kanekura et al., 2006). Interestingly, Simmen et al. (2005) have shown that loss of ER/mitochondria contacts induces ER stress and the UPR, probably by disturbing some of the ER chaperones together with calnexin, calreticulin, and Sigma non-opioid intracellular receptor 1 (Sig1R; Hayashi and Su, 2007).

## TDP-43 and ER Stress and UPR

Aberrant misfolding and aggregation of TDP-43 along with TDP-43 posttranslational modifications play a crucial role in the development of neurodegenerative diseases including ALS and FTD (Arai et al., 2006; Neumann et al., 2006; Igaz et al., 2008; Kumar et al., 2016b; 2019). Also, pathological aggregation of TDP-43 causes increased ER stress following the activation of apoptosis (Suzuki et al., 2011; Dafinca et al., 2021). Nuclear depletion of TDP-43 correlates with increased mislocalization of TDP-43 to the rough ER and cytoplasmic inclusion formation, indicating a dynamic relationship between the TDP-43, ER, and pathological inclusion leading to neurodegeneration (Sasaki, 2010; Vaccaro et al., 2013; Wang et al., 2015a). Later, Suzuki and Matsuoka (2012) reported that the increased endogenous levels of TDP-43 result with the increase in the expression of C/EBP-homologous protein(CHOP), largely involved in ER-facilitated apoptosis and is regulated *via* the PERK/eIF2a/ATF4 pathway. Walker et al. (2013) showed that both WT and mutant TDP-43^Q331K/A315T^ significantly activate the UPR pathways through increasing the expression of the key ER stress markers, XBP1 and ATF6 in Neuro2a cells. Moreover, overexpression of TDP-43 C-terminal fragments, TDP-35, and TDP-25 increased the levels of phosphorylated-eIF2α, CHOP, and truncated caspase-12 (Wang et al., 2015b). These findings were further established by Wang et al. (2015a), who showed that overexpression of WT and TDP-43^A315T^ in neural SH-SY5Y cells up-regulates the expression of the GRP-78, phosphorylated-eIF2α, CHOP, caspase-3, caspase-9, and caspase-12 fragments, as well as downregulates the Bcl-2 family proteins. Similarly, Hu et al. (2019) showed that overexpression of mutant TDP-43^Q331K^ in SH-SY5Ycellsleads to increased levels of GRP-78, ATF4, CHOP, PDI, and caspase 12. Also, TDP-43^Q331K^mutant enhanced Beclin1 and p62 expression, and decreased the LC3-II/LC3-I ratio, an indicative of impaired autophagy normally observed in several neurodegenerative diseases.

## Pharmacological Targeting of ER Stress and UPR

The fact that ER stress is involved in ALS pathogenesis and the UPR pathway which mitigates ER stress under physiological conditions represents a key therapeutic target for interventions. The activation of the UPR signaling pathway can lead to the activation of both pro-survival and pro-apoptotic activities. Thus, modulating the UPR pathways will either stimulate alleviation of protein misfolding, or stimulate apoptosis, which would have therapeutic effects in human diseases associated with ER stress. Many studies have identified small molecules that can be considered as a potential drug to selectively inhibit UPR components and have already been enrolled in preclinical trials of disease (Kanekura et al., 2009; Saxena et al., 2009; Ciechanover and Kwon, 2015; Ruegsegger and Saxena, 2016; Dalla Bella et al., 2021).

Several small molecules targeting the different domains of the IRE1 pathway are in the preclinical stages against cancer, diabetes, neurodegenerative diseases, etc. The RNase domain inhibitors of IRE1 (e.g., MKC-3946, STF-083010) contain an aromatic aldehyde group in the core and adjacent hydroxyl group. The core aldehyde group reacts with the amino group of lysine and forms imine derivatives, and the hydroxyl group reacts with the tyrosine forming hydrogen bonds, and inhibiting the catalytic cleavage of IRE1α (Cross et al., 2012). The inhibitors targeting the kinase domain of IRE1 (e.g., KIRA6) disrupt the interfacial contacts and prevent the dimerization of IRE1α, thus making it inactive (Feldman et al., 2016). The small molecule inhibitors of the PERK pathway (e.g., GSK2606414, GSK2656157) prevent the interaction of PERK with ATP and thus block the PERK induced signaling cascade. These ATP-competitive inhibitors contain an indoline core moiety that binds to the kinase domain in the ATP-binding site, leading to a conformational change that results in the inhibition of the kinase domain of PERK (Hetz et al., 2019). Similarly, the modulators of the PERK pathway (Salubrinal, Guanabenz, and Sephin1) inhibit the protein phosphatase complex and thus prevent the de-phosphorylation of eIF2α, a negative feedback loop to control protein translation during the ER stress (Boyce et al., 2005; Tsaytler et al., 2011). Also, Salubrinal protects SOD1^G93A^ mouse motor neurons from ER stress (Saxena et al., 2009). Guanabenz, an FDA-approved alpha-2 adrenergic receptor agonist, decreased neuronal toxicity by decreasing the ER stress in worm and zebrafish models (Vaccaro et al., 2013). Guanabenz treatment delayed the disease onset, improved motor performance, decrease the loss of motor neurons, and improve the survival in a SOD1^G93A^ mouse model by reducing ER stress due to extensive phosphorylation ofeIF2a (Jiang et al., 2014; Wang et al., 2014; Das et al., 2015; Dalla Bella et al., 2021). In mutant TDP-43 *C. elegans* and zebrafish models of ALS, guanabenz, and salubrinal decreased ER stress and subsequently reduced the neurodegeneration (Vieira et al., 2015).

A summary of small molecules that selectively target crucial UPR components and other players of the ER proteostasis network, and is used to target neurodegenerative disease models is elegantly reviewed in Charif et al. (2022), Hetz et al. (2019), and Rivas et al. (2015) and is further shown in Figure 1 and Table 1.

**Table 1:** Pharmacological targeting of UPR pathway (Rivas et al., 2015; Hetz et al., 2019).

**Small molecules**	**UPR pathway**	**ER-stress**
GSK2656157	PERK arm	Inhibitor of PERK kinase
GSK2606414		Inhibitor of PERK kinase
Salubrinal		Binding GADD34 phosphatase complex, inhibitor of eIF2a dephosphorylation
ISRIB		Reduced ATF4 expression
Guanabenz		eIF2a phosphatase inhibitor
Sephin1		eIF2a phosphatase inhibitor
Salicylaldimines	IRE1 arm	Inhibitor of IRE1α RNase
SFT-083010		Inhibitor of IRE1α RNase
MKC-3946		Inhibitor of IRE1α RNase
Sunitinib		Inhibitor of IRE1α RNase
Toyocamycin		Inhibitor of IRE1α RNase
Methoxycitrinin		Increasing the XBP1 splicing levels
Citrinin		Increasing the XBP1 splicing levels
Patulin		Increasing the XBP1 splicing levels
Quercetin		Increase IRE1 nuclease activityand splicing of XBP1
Apigenin		Increasingthe IRE1α nuclease activity
Resveratrol		Decreasing DNA-binding capacity of XBP1 to the target genes
Apigenin	ATF6 arm	Upregulation of ATF6 expression
Baicalein		Upregulation of ATF6 expression
Kaempferol		Downregulation of ATF6 expression

## Conclusion

In summary, growing evidences indicate that ER stress and UPRare largely involved in the pathogenesis of human diseases, including cancer and neurodegenerative diseases. Protein misfolding and aggregation in cells trigger the ER stress which is overpowered by an adaptive response of cells collectively known as UPR. The UPR pathways are facilitated by essential components like PERK, IRE1, and ATF6 which are important in maintaining protein homeostasis. This protective role becomes more important and beneficial in several neurodegenerative diseases including ALS. Studies from ALS and other neurodegenerative disease models indicate that treatments targeting ER proteostasis and UPR have shown protective roles. Further, a more clear and improved understanding of the genes involved in ALS and the associated mechanisms of proteostasis dysfunction in ALS will be essential and crucial for developing small molecule therapeutics to effectively target the ER proteostasis and UPR.

## Author Contributions

VK and YL designed the topics. CZ and AR wrote the main text with the contributions of YL and VK. All authors contributed to the article and approved the submitted version.

## Conflict of Interest

The authors declare that the research was conducted in the absence of any commercial or financial relationships that could be construed as a potential conflict of interest.

## Publisher’s Note

All claims expressed in this article are solely those of the authors and do not necessarily represent those of their affiliated organizations, or those of the publisher, the editors and the reviewers. Any product that may be evaluated in this article, or claim that may be made by its manufacturer, is not guaranteed or endorsed by the publisher.
